# Gender-based violence and its association with mental health among Somali women in a Kenyan refugee camp: a latent class analysis

**DOI:** 10.1136/jech-2020-214086

**Published:** 2020-11-03

**Authors:** Mazeda Hossain, Rachel Jane Pearson, Alys McAlpine, Loraine J Bacchus, Jo Spangaro, Stella Muthuri, Sheru Muuo, Giorgia Franchi, Tim Hess, Martin Bangha, Chimaraoke Izugbara

**Affiliations:** 1 Department of Global Health & Development, London School of Hygiene & Tropical Medicine, London, UK; 2 Centre for Women, Peace & Security, London School of Economics and Political Science, London, UK; 3 Population, Policy and Practice Research and Teaching Department, UCL Great Ormond Street Institute of Child Health, London, UK; 4 School of Health and Society, University of Wollongong, Wollongong, Australia; 5 African Population and Health Research Center, Nairobi, Kenya; 6 Violence Prevention and Response Unit, International Rescue Committee UK, London, UK

**Keywords:** Disaster relief, Gender, Health services, Mental health, Violence

## Abstract

**Background:**

In conflict-affected settings, women and girls are vulnerable to gender-based violence (GBV). GBV is associated with poor long-term mental health such as anxiety, depression and post-traumatic stress disorder (PTSD). Understanding the interaction between current violence and past conflict-related violence with ongoing mental health is essential for improving mental health service provision in refugee camps.

**Methods:**

Using data collected from 209 women attending GBV case management centres in the Dadaab refugee camps, Kenya, we grouped women by recent experience of GBV using latent class analysis and modelled the relationship between the groups and symptomatic scores for anxiety, depression and PTSD using linear regression.

**Results:**

Women with past-year experience of intimate partner violence alone may have a higher risk of depression than women with past-year experience of non-partner violence alone (Coef. 1.68, 95% CI 0.25 to 3.11). Conflict-related violence was an important risk factor for poor mental health among women who accessed GBV services, despite time since occurrence (average time in camp was 11.5 years) and even for those with a past-year experience of GBV (Anxiety: 3.48, 1.85–5.10; Depression: 2.26, 0.51–4.02; PTSD: 6.83, 4.21–9.44).

**Conclusion:**

Refugee women who experienced past-year intimate partner violence or conflict-related violence may be at increased risk of depression, anxiety or PTSD. Service providers should be aware that compared to the general refugee population, women who have experienced violence may require additional psychological support and recognise the enduring impact of violence that occurred before, during and after periods of conflict and tailor outreach and treatment services accordingly.

## INTRODUCTION

The number of people forcibly displaced due to conflict, violence and persecution has increased by more than 60% since 2009, reaching 70.8 million people in 2018 of which 25.9 million of this group were refugees.^1^ In conflict-affected settings, women and girls are particularly vulnerable to gender-based violence (GBV). GBV is not only linked to adverse health outcomes, but also affects women’s ability to secure and undertake employment and their participation in recovery efforts.^2^ Programmes that prevent and respond to GBV against women in populations affected by conflict are, therefore, critical for reducing gender inequity and improving population health and economic growth in these settings.

Until recently, programming and guidance for responses to GBV within displaced populations were largely concentrated on sexual violence.^3^ Historically, rape has been weaponised during conflict to terrorise and control and, in settings of genocide, to aid ethnic cleansing.^4–6^ Women are also extremely vulnerable to sexual exploitation and sexual slavery in times of conflict.^7^ Post conflict, displaced women are at risk of experiencing further sexual violence while in transit and within refugee camps or host-communities.^8^ However, there is growing evidence that women in conflict-affected settings face many other forms of GBV. For example, intimate partner violence (IPV) is estimated to be more prevalent among conflict-affected populations than GBV perpetrated by non-partners.^9 10^ Similarly, harmful practices including early/forced marriage and female genital mutilation may increase during conflict although research on how conflict may influence these practices is limited.^11^

GBV against women and girls is linked to many poor health outcomes including long-term mental health disorders such as anxiety, depression and post-traumatic stress disorder (PTSD).^2 12^ In conflict-affected settings, both recurrent episodes of violence and conflict-related sexual violence have been linked to PTSD and some forms of IPV are linked to an increased risk of anxiety and depression.^13–16^ GBV has also been associated with adverse physical and psychosocial outcomes that may contribute to mental health problems such as unwanted pregnancies, sexually transmitted infections and discrimination, stigmatisation and ostracism within the family or community.^17 18^ However, there is a scarcity of evidence on the spectrum of GBV experienced in conflict-affected settings and whether mental health outcomes differ between women experiencing different forms of GBV. Therefore, to develop effective prevention and response programmes and strategies for this population, a better understanding of the relationship between differing experiences of GBV and mental health is needed.

We explored some of these issues in our research in the Dadaab refugee camp complex. The Dadaab refugee camps in Garissa County, Kenya were established in 1992 in response to those fleeing the Somali civil war and formed one of the largest refugee camps in the world, hosting over 345 000 people in 2016.^19^ Most (95%) of the residents have Somali nationality, with the remainder from neighbouring countries including Ethiopia and South Sudan who were driven by long-term drought, economic migration and conflict. Due to prolonged regional insecurity and conflict, drought and famine in Central and East Africa, Dadaab has continued to experience an influx of refugees from Somalia and other countries. Similar to many other refugee camps, it is characterised by a lack of adequate access to basic amenities, food and water, and poor sanitation, living and economic conditions. In Dadaab’s two largest camps, Dagahaley and Hagadera, the International Rescue Committee (IRC) and CARE International Kenya (CIK) have implemented a case management intervention to assist survivors of GBV.^20^ Using a task-sharing approach, IRC and CIK employ refugee community workers to improve the breadth of support that the case management model is able to offer, including accompaniment to referrals, translation between survivors and national staff and informal counselling.

Recognising the likely interaction between current experiences of GBV and past conflict-related violence with mental health, we sought to understand the types of violence that women living in two of the Dadaab refugee camp complex have experienced and the relationship between those experiences of violence and current mental health symptomology. Understanding these differences may help GBV and mental health service providers in refugee camp settings to tailor their support services based on women’s past and current experiences of violence. To inform the development of the GBV case management service model in Dadaab, we posed the question—how does mental health symptomology differ between female survivors of different types of violence living in the Dadaab refugee camps? Latent class analysis (LCA) has previously been used to identify and understand patterns of GBV experiences among conflict-affected populations.^15 21^ Here, we used LCA to group women with similar experiences of non-partner violence (NPV) and IPV in the 12 months before intake to GBV services in Dadaab, to explore whether risk of mental health problems (anxiety, depression and PTSD) differed between groups.

## METHODS

### Study design

We used baseline data previously collected within a prospective cohort study, jointly led by the London School of Hygiene and Tropical Medicine (LSHTM) and the African Population and Health Research Centre (APHRC), in collaboration with IRC and CIK.^20^ Between February and November 2016, women older than 18 years were recruited from centres in Dagahaley and Hagadera which offered the IRC/CARE case management model; girls between the ages of 15–18 who were either married or the sole head of their household were also eligible to participate, though none did. Eligibility was assessed by each centre’s national staff. A total of 209 women (132 from Hagadera, 77 from Dagahaley) participated and subsequently completed the baseline questionnaire conducted by a trained member of the research field team. The questionnaires were translated from English to Somali and interviews could be conducted in either language. All procedures involving human subjects/patients were approved by the LSHTM local ethics committee (8909) and the Scientific Review Committee of the African Medical and Research Foundation (AMREF) in 2015.

### Gender-based violence

Experiences of GBV were captured in the baseline questionnaire. Seventeen items, adapted from the WHO’s multi-country study on women’s health and domestic violence against women, were used to determine emotional, physical and sexual IPV and physical and sexual NPV within several time periods of interest (table 1).^22^ Past-year IPV was identified among ever-partnered women in the cohort who were asked whether their current or most recent partner had perpetrated specific acts of emotional, physical and sexual violence against them in the previous 12 months. Past-year NPV was identified among all members of the cohort who were asked whether they had experienced specific acts of physical or sexual violence, perpetrated by non-partners, in the previous 12 months. Participants were also asked if they had experienced NPV before they arrived at the camps.

**Table 1:** Gender-based violence types: measuring intimate-partner violence and non-partner violence among women accessing GBV centres in two of the Dadaab camps

Type of gender-based violence	Subtype	Question
**Intimate Partner Violence**	Emotional Violence	Has your (current/most recent partner) ever become angry when you speak to other men? Does your (current/most recent) partner insist on knowing where you were at all times? Does your (current/most recent) partner ever forbid you from seeing your friends? Has your partner ever done something to frighten or intimidate you? (eg, in the way she/he looks at you or by yelling or breaking something?) Has your partner ever threatened to hurt you or someone you care about?
	Physical Violence (moderate)*	Has your partner ever slapped you or thrown something that could hurt you? Has your partner ever pushed or shoved you? Has your partner ever hit you with his hand or with something else that could hurt you?
	Physical Violence (severe)†	Has your partner ever kicked, dragged or beaten you? Has your partner choked you or burned you intentionally? Has your partner THREATENED to use a gun, knife or other weapon against you? Has your partner ACTUALLY used a gun, knife or other weapon against you?
	Sexual Violence	Has your partner ever forced you to have sex by using threats or intimidation (not physical violence)? Has your partner ever physically forced you to have sex when you did not want to?
**Non-partner violence**	Physical Violence	Has anyone who is not your intimate partner ever beaten you with a fist, or kicked you, or hurt you with a stick or other object? Has anyone who is not your intimate partner ever used a gun, knife or other weapon against you?
	Sexual Violence	Has anyone who is not your intimate partner ever forced you to have sex when you did not want to, for example by threatening you, holding you down, or putting you in a situation where you could not say no?

*Moderate physical IPV has occurred when a woman reports more than one event of any moderate physical act of IPV.

†Severe physical IPV has occurred when a woman reports at least one event of any severe physical act of IPV. GBV, gender-based violence; IPV, intimate partner violence.

### Mental health outcomes

Our three primary outcomes were probable anxiety, depression and PTSD in the two weeks prior to interview. These were measured using symptomatic scales, each scored on a 4-point Likert scale, with higher scores indicating greater severity. Anxiety was measured via the Generalized Anxiety Disorder-7 (GAD-7) scale, a 7-item anxiety scale used to screen for generalised anxiety disorder, where cut-off scores of 5, 10 and 15 are recommended for mild, moderate and severe anxiety, respectively.^23^ Depression was measured by the Patient Health Questionnaire-9 (PHQ-9), a 9-item scale based upon the depression criteria from the Diagnostic and Statistical Manual for Mental Disorders, fourth edition (DSM-IV).^24^ Cut-off scores of 5, 10, 15 and 20 are recommended for mild, moderate, moderately severe and severe depression, respectively. Previously, the PHQ-9 has been translated to Somali for use among Somali immigrants in the United States (US) and demonstrated good internal validity.^25^ PTSD was measured using the post-traumatic symptom subscale of the Harvard Trauma Questionnaire (HTQ-PTSD).^26^ The HTQ-PTSD is a 16-item sub-scale derived from the DSM-IV PTSD criteria. This screening tool for probable PTSD, developed for adult refugees, has been validated within several refugee populations.^13^ It has also previously been used among Somali refugees, demonstrating good internal validity.^27^ A mean score cut-off of 2 (from a theoretical range of 1–4) is recommended for probable PTSD.^26^ Each of these symptomatic scales demonstrated good internal validity (anxiety: Cronbach’s α =0.77, depression: Cronbach’s α =0.77 and PTSD: Cronbach’s α =0.83).

### Socio-demographic characteristics

The baseline questionnaire captured several socio-demographic variables including age at interview, average monthly income in Kenyan shillings (KES), length of encampment, number of children cared for, literacy status, nationality, religion, partner status and belonging to a majority Somali clan (ie, the Darod, Dir, Hawiye or Isaaq clans).

### Statistical analysis

We derived descriptive statistics (ie, frequencies and percentages or, for continuous variables, the median and IQR) for sociodemographic characteristics, GBV measures and probable anxiety, depression and PTSD.

We used latent class analysis (LCA) with maximum probability assignment to group women by their experiences of past-year GBV. Using the indicators for emotional IPV, moderate physical IPV, severe physical and/or sexual IPV and physical and/or sexual NPV, we derived latent groupings for patterns of past-year GBV observed in the data. We considered models with up to four classes and increased the number of sets of random starting values with model complexity to improve model convergence to the global maximum likelihood solution. Robust SEs, which produce optimal SEs when only parts of the model are correctly specified, were calculated for each model.^28^ We evaluated relative goodness-of-fit between models, using the Akaike information criterion (AIC) and the Bayesian information criterion (BIC), with lower values indicating better model fit.^28^ We also used the odds of correct classification (OCC) to assess the appropriateness of maximum probability assignment, where an OCC of at least 5 for each class indicates good latent class separation (ie, assignment is better than chance).^28 29^

We modelled the associations between the latent groupings and each outcome (anxiety, depression and PTSD) using fixed-effects linear regression models, with camp-specific intercepts to account for clustering by camp. Models were adjusted for age, average monthly income, length of encampment, number of children, partner status, belonging to a majority clan and any experience of NPV before arrival to Dadaab, selected a priori, as each are important predictors of the outcomes.^30^

We conducted a secondary analysis using indicator variables for past-year IPV and past-year NPV in place of the latent grouping variable to compare against the main findings. Again, adjusted fixed-effects models were fitted for each symptomatic scale.

All study measures had complete data, except length of encampment which was missing for five women. We conducted a complete case analysis; therefore, the fixed-effects linear regression models used data from the 204 participants with complete data. Model assumptions and fit were checked using residual plots and diagnostic statistics including the R-squared and the root mean square error (RMSE). Given the small sample size, our capacity to conduct statistical hypothesis tests was limited; therefore, we discuss the statistical significance of the findings, the direction and strength of the effects and the broader implications of the findings. All data management and analyses were conducted using Stata v15.

## RESULTS

### Sociodemographic characteristics

At the time of interview, participants were aged between 18 and 66 years old, with a mean age of 29 years (table 2). The mean length of stay in Dadaab among the cohort was 11.5 years and 9% of the participants were born within the camps. Most participants had Somali nationality (94%), belonged to one of the four majority Somali clans (62%) and were Muslim (99%). Forty-five per cent of the participants had a current intimate partner who they lived with and 41% of the participants cared for at least four children. Almost two-thirds of participants (61%) had little to no monthly income (0–4500 KES) and only 36% of participants reported being able to both read and write.

**Table 2:** Socio-demographic characteristics and mental health among participants, recorded at intake to Gender-Based Violence (GBV) services

	Dagahaley (N=77) %	Hagadera (N=132) %	Total (N=209) %
Age (years)			
18–24	34 (44.2)	44 (33.3)	78 (37.3)
25–34	31 (40.3)	51 (38.6)	82 (39.2)
35–44	10 (13.0)	25 (18.9)	35 (16.7)
45+	2 (2.6)	12 (9.1)	14 (6.7)
Length of encampment (years)			
Born in Dadaab	12 (15.6)	6 (4.5)	18 (8.6)
0–10	37 (48.1)	71 (53.8)	108 (51.7)
10+	23 (29.9)	55 (41.7)	78 (37.3)
Missing	5 (6.5)	0 (0.0)	5 (2.4)
Nationality			
Somalian	71 (92.2)	126 (95.5)	197 (94.3)
Other	6 (7.8)	6 (4.5)	12 (5.7)
Majority Somali clan*			
No	27 (35.1)	52 (39.4)	79 (37.8)
Yes	50 (64.9)	80 (60.6)	130 (62.2)
Religion			
Muslim	74 (96.1)	132 (100.0)	206 (98.6)
Christian	3 (3.9)	0 (0.0)	3 (1.4)
Partner status			
Partner absent/not partnered	42 (54.5)	73 (55.3)	115 (55.0)
Partner present	35 (45.5)	59 (44.7)	94 (45.0)
Number of children			
None	14 (18.2)	28 (21.2)	42 (20.1)
1–3	32 (41.6)	50 (37.9)	82 (39.2)
4+	31 (40.3)	54 (40.9)	85 (40.7)
Able to read and write			
No	36 (46.8)	93 (70.5)	129 (61.7)
Yes	41 (53.2)	39 (29.5)	80 (38.3)
Monthly income (KES)			
0–4499	61 (79.2)	66 (50.0)	127 (60.8)
4500–9999	14 (18.2)	37 (28.0)	51 (24.4)
10 000+	2 (2.6)	29 (22.0)	31 (14.8)
Mental health			
Moderate to severe anxiety (GAD-7)	24 (31.2)	61 (46.2)	85 (40.7)
Moderate to severe depression (PHQ-9)	10 (13.0)	65 (49.2)	75 (35.9)
Probable PTSD symptomology (HTQ-PTSD)	0	6 (4.5)	6 (2.9)
At least one of the above	28 (36.4)	79 (59.8)	107 (51.2)

*Majority clan is defined here as one of the following Somali clans: Darod, Dir, Hawiye, Isaaq.

GAD-7, Generalized Anxiety Disorder-7 scale; HTQ, Harvard Trauma Questionnaire; KES, Kenyan shillings; PHQ-9, Patient Health Questionnaire-9;PTSD, Post-traumatic stress disorder.

### Mental health outcomes

Poor mental health was highly prevalent among women accessing services. Over half of the participants had symptomatic scores (51%), including scores indicative of moderate to severe anxiety (41%), depression (36%), or probable PTSD (3%). However, the prevalence of at least one of these was much lower among those attending the centre in Dagahaley (36%) than in Hagadera (60%) (table 2).

### Experiences of gender-based violence

**Figure 1:**
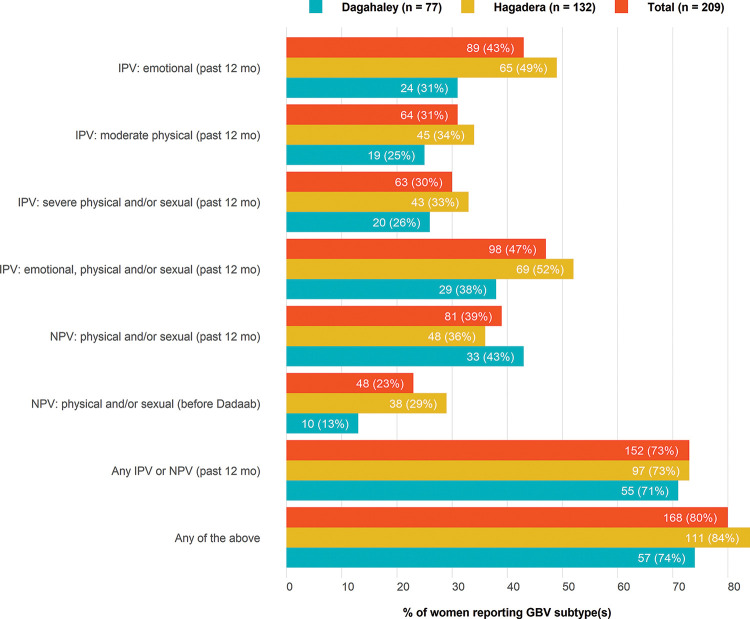
Baseline prevalence of gender-based violence, by type of violence, time period and camp among women accessing services in a refugee camp. GBV, Gender-Based Violence; IPV, Intimate Partner Violence; NPV, Non-Partner Violence.

The most reported form of past-year GBV was IPV (figure 1). Almost half (47%) of the participants reported IPV in the year prior to interview, 63% of which reported two or more forms of IPV (data not displayed). Almost all (91%) of the participants reporting past-year IPV experienced emotional violence by their intimate partner. Fewer participants reported NPV in the past year (39%), among which 93% reported physical violence and 16% reported sexual violence. Similarly, only 23% of participants reported experiences of NPV before their arrival to Dadaab refugee camp, with 92% reporting physical violence and 31% reporting sexual violence.

### Grouping experiences of past-year gender-based violence

**Figure 2:**
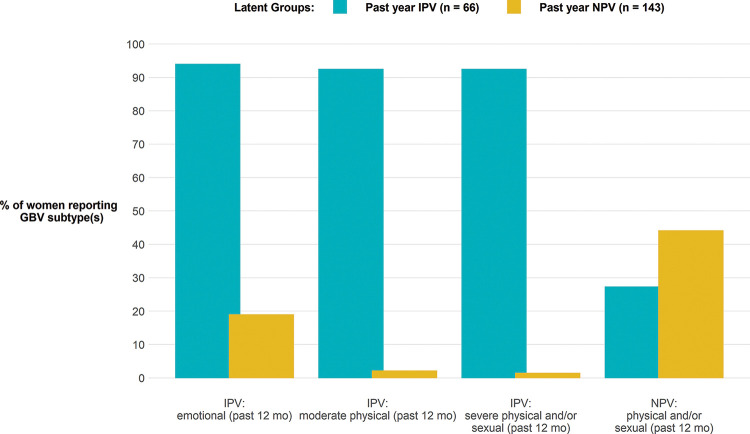
Latent class analysis grouping of past-year experiences of gender-based violence among refugee women accessing services. GBV, Gender-Based Violence. IPV, Intimate Partner Violence; NPV, Non-Partner Violence.

Based on interpretability of the latent classes, the AIC and BIC, and the OCC, we chose the two-class latent model as our final LCA model. The first class (the past-year IPV class) was clearly defined by the past-year emotional IPV, past-year moderate physical IPV and past-year severe physical and/or sexual IPV items (n=66, 32%), while the other class (the past-year NPV class) was defined by past-year physical and/or sexual NPV item (n=143, 68%) (figure 2).

### Primary analysis: modelling the association between the latent groupings of past-year gender-based violence and mental health

The adjusted fixed-effects model for the symptomatic scores for depression showed evidence that participants in the past-year IPV latent group had higher symptomatic scores for depression than participants in the past-year NPV latent group (−1.68, 95% CI: 0.25 to −3.11), holding the other model covariates constant (table 3). However, both the fixed-effects models for anxiety and PTSD provided no evidence that symptomatic scores differed between women in either latent group. Experience of NPV before Dadaab was strongly associated with higher scores for anxiety (3.48, 1.85 to 5.10), depression (2.26, 0.51 to 4.02) and PTSD (6.83, 4.21 to 9.44), adjusted for the other model covariates.

**Table 3:** Primary model results: modelling the association between past-year GBV latent groupings and mental health symptomatic scores anxiety, depression and post-traumatic stress disorder

	(1)			(2)			(3)		
	PHQ-9 score (Depression)			GAD-7 score (Anxiety)			HTQ-PTSD score (Post-Traumatic Stress)		
	Coef	95% CI	P value	Coef	95% CI	P value	Coef	95% CI	P value
Past-year NPV latent class (Ref)	0.00			0.00			0.00		
Past-year IPV latent class	1.68	[0.25, 3.11]	0.021	0.91	[−0.49, 2.32]	0.201	0.58	[−1.82, 2.97]	0.635
Age (years), mean centred	−0.02	[−0.10, 0.07]	0.696	−0.09	[−0.18, −0.01]	0.027	−0.06	[−0.17, 0.06]	0.326
Majority clan*: No (Ref)	0.00			0.00			0.00		
Majority clan*: Yes	0.64	[−0.70,1.98]	0.350	−0.40	[−1.70, 0.90]	0.547	−0.84	[−3.03, 1.34]	0.448
Read/write: No (Ref)	0.00			0.00			0.00		
Read/write: Yes	−0.17	[−1.52, 1.18]	0.801	0.54	[−0.87, 1.95]	0.450	1.36	[−0.97, 3.69]	0.250
Length of encampment (years)	−0.03	[−0.13, 0.06]	0.487	0.03	[−0.05, 0.12]	0.451	0.01	[−0.13, 0.14]	0.933
Number of children	−0.04	[−0.29, 0.22]	0.763	0.25	[−0.00, 0.51]	0.053	0.17	[−0.22, 0.55]	0.392
Monthly income (KES)	0.00	[0.00, 0.00]	0.812	0.00	[0.00, 0.00]	0.272	0.00	[0.00, 0.00]	0.165
any NPV before Dadaab: No (Ref)	0.00			0.00			0.00		
any NPV before Dadaab: Yes	2.26	[0.51, 4.02]	0.012	3.48	[1.85, 5.10]	<0.001	6.83	[4.21, 9.44]	<0.001
R^2^	0.77			0.79			0.82		
RMSE	4.52			4.47			7.16		

*Majority clan is defined here as one of the following Somali clans: Darod, Dir, Hawiye, Isaaq.

GBV, Gender-Based Violence; IPV, Intimate Partner Violence; KES, Kenyan Shillings; NPV, Non-Partner Violence; RMSE, Root mean square error.

### Secondary analysis: modelling the association between indicators of GBV and mental health

There was evidence that all three victimisation exposures were associated with an increase in the symptomatic scores for depression among participants; past-year IPV (1.39, 0.07 to 2.70), past-year NPV (1.28, −0.09 to 2.64) and NPV before Dadaab (2.09, 0.34 to 3.84) (table 4). NPV before Dadaab was also strongly associated with higher symptomatic scores for anxiety (3.31, 1.64 to 4.97) and PTSD (6.51, 3.84 to 9.18), adjusted for other model covariates.

**Table 4:** Secondary model results: modelling the association between indicators of GBV and mental health symptomatic scores for anxiety, depression and post-traumatic stress disorder

	(1)			(2)			(3)		
	PHQ-9 score			GAD-7 score			HTQ-PTSD score		
	Coef	95% CI	P value	Coef	95% CI	P value	Coef	95% CI	P value
Age (years), mean centred	−0.01	[−0.10, 0.07]	0.737	−0.09	[−0.18, −0.01]	0.025	−0.05	[−0.17, 0.07]	0.377
Majority clan: No (Ref)	0.00			0.00			0.00		
Majority clan: Yes	0.63	[−0.72, 1.97]	0.360	−0.38	[−1.69, 0.93]	0.572	−0.84	[−3.04, 1.36]	0.452
Read/write: No (Ref)	0.00			0.00			0.00		.
Read/write: Yes	−0.40	[−1.71, 0.92]	0.553	0.36	[−1.03, 1.74]	0.613	1.24	[−1.00, 3.48]	0.278
Length of encampment (years)	−0.05	[−0.14, 0.04]	0.299	0.02	[−0.06, 0.11]	0.591	−0.01	[−0.14, 0.12]	0.888
Number of children	−0.04	[−0.30, 0.22]	0.768	0.25	[0.00, 0.51]	0.054	0.17	[−0.21, 0.55]	0.374
Monthly income (KES)	0.00	[0.00, 0.00]	0.740	0.00	[0.00, 0.00]	0.275	0.00	[0.00, 0.00]	0.172
Past-year IPV: No (Ref)	0.00			0.00			0.00		
Past-year IPV: Yes	1.39	[0.07, 2.70]	0.039	0.39	[−0.95, 1.74]	0.565	0.42	[−1.80, 2.65]	0.707
Any NPV before Dadaab: No (Ref)	0.00			0.00			0.00		
Any NPV before Dadaab: Yes	2.09	[0.34, 3.84]	0.020	3.31	[1.64, 4.97]	<0.001	6.51	[3.84, 9.18]	<0.001
Past-year NPV: No (Ref)	0.00			0.00			0.00		
Past-year NPV: Yes	1.28	[−0.09, 2.64]	0.066	0.70	[−0.63, 2.03]	0.303	1.54	[−0.61, 3.69]	0.160
R^2^	0.77			0.79			0.82		
RMSE	4.53			4.49			7.15		

*Majority clan is defined here as one of the following Somali clans: Darod, Dir, Hawiye, Isaaq.

GAD-7, Generalized Anxiety Disorder-7 scale; GBV, gender based violence; HTQ, Harvard Trauma Questionnaire; IPV, Intimate Partner Violence; KES, Kenyan Shillings; NPV, Non-Partner Violence; PHQ-9, Patient Health Questionnaire-9; PTSD, Post-traumatic stressdisorder; RMSE, Root mean square error.

## DISCUSSION

### Main findings

Our analysis demonstrates the multiple avenues leading to poor mental health faced by women in refugee camps. Women accessing services in our study reported poor mental health from both current experiences of violence (IPV) and past experiences (NPV during the conflict). Importantly, we found that women presenting with past year experience of IPV may be at a greater risk of depression than those presenting with past year experience of NPV. We also found that conflict-related violence was an important risk factor for poor mental health among women who accessed GBV services, despite time since occurrence (average time in camp was 11.5 years) and even among those who had a past year experience of GBV. Similar proportions of study participants reported past-year intimate partner violence (IPV) as reported past-year non-partner violence (NPV). Among those reporting past-year IPV, over half experienced two or more forms (emotional, physical and sexual) and almost all experienced emotional IPV. Almost a quarter of participants reported physical or sexual violence by a non-partner before arrival to Dadaab.

These findings suggest that the GBV centres in Dadaab are reaching women with experiences of particularly severe and systematic IPV, and also highlight the need for GBV programming that caters to the needs of survivors of IPV as well as to violence perpetrated outside of an intimate relationship including conflict-related violence. The WHO estimate that 3–4% of refugees may suffer from severe mental health disorders, while 15–20% may suffer from mild/moderate disorders;^31^ however, we found that more than half of the study participants reported symptoms of either probable PTSD or moderate/severe depression or anxiety. This suggests that there is a high prevalence of mental health disorders among women who experience GBV in conflict-affected settings in comparison to the wider refugee population.

Modelling the relationship between the latent GBV groupings and anxiety, depression and PTSD suggested that women with recent experience of IPV alone may have a higher risk of depression than women with past-year experience of NPV alone. These results are similar to those from a cross-sectional study among Congolese women living in Rwandan refugee camps where women in the ‘High IPV’ latent class were found to have twice the odds of experiencing emotional distress than those in the NPV during or after conflict class.^15^ In addition, our secondary analysis showed evidence that an experience of any form of IPV in the past year was associated with higher scores for depression, even after adjustment for past-year NPV.

We also found strong evidence that women who experienced physical and/or sexual NPV before Dadaab had higher symptomatic scores for all three mental health scales than those who did not, despite an average length of encampment among the cohort of 11.5 years. As 95% of the cohort are Somali, it is probable that many of the recorded physical and/or sexual acts committed by a non-partner before Dadaab were perpetrated during the Somali civil war, which began in the early 1990s, and ongoing conflict thereafter. This suggests that the negative effects of these traumatic conflict-related events on a survivor’s mental health can be long-lasting.

### Strengths and limitations

A key strength of this study is the breadth and depth of information collected which allowed us to adjust models for a range of potentially confounding baseline characteristics. Another strength is the measures used. In particular, the PHQ-9 and HTQ-PTSD had previously been used among Somali refugees demonstrating good internal validity.^25 27^ We also used a set of indicators for GBV that had been used in multiple previous studies, allowing for comparisons.

Despite these strengths, interpretation of the findings must consider several caveats. Initially, the study set out to recruit 400 women but ultimately only 209 women were enrolled in the study. During the study period, the Kenyan government announced its intention to close the Dadaab camps which resulted in a sharp increase in refugee repatriation. To date, two of the five camps within the Dadaab camp complex have since closed. In addition to the departure of many refugees from the camps, repatriation-related activities within the camps likely diverted resources away from services relied upon by the GBV centres to reach survivors of GBV and involvement in repatriation processes may also have prevented women from attending centres. It is likely that the threatened camp closure exacerbated existing mental health problems, affecting the specificity of the symptomatic scale for anxiety.

The small sample size was a particularly restricting factor for the LCA. Reporting of most individual items of GBV was low; therefore, some items were aggregated. It is possible that, with a larger sample, more subtle patterns of GBV beyond a two-class solution may have been detected.

A further limitation was the perpetrator types described in the questionnaire. There were 24 women (11%) with no reported lifetime GBV. However, the free-text responses to ‘reason for service visit’ in the interviews revealed that many were accessing the GBV services in response to threats and intimidation from within the community or their own families. A recent study among women in rural Côte d’Ivoire suggests that in-law abuse may be more prevalent among women who experienced personal adversity during conflict or whose families experienced conflict-related violence.^32^ Reporting of GBV, sexual violence in particular, was also likely affected by response bias for many reasons including the stigma attached to these forms of violence and differing cultural norms on what is and what is not recognised as consensual sex.^20 22^

Finally, we were unable to compare characteristics between women attending the GBV centres over the study period who participated in the study and those who did not. To reduce impact on staff and service provision, staff were not required to record information on women who did not participate. We also lacked demographic information on all women living in either camp; therefore, we could not ascertain whether differences in sociodemographic characteristics, experiences of GBV and mental health symptomology were due to differences between the camps or differences in the way women were selected for participation between the GBV centres. In addition, women suffering from severe mental illness, severe physical or cognitive disability, or experiencing severe forms of GBV may face more barriers to accessing care at the centres; hence, the most extreme cases of GBV and/or poor mental health may not have been captured in the cohort which may underestimate the strength of the associations found.

### Implications

While not generalisable to all refugee camp settings, this investigation adds to the growing list of research concluding that in conflict-affected settings, IPV has adverse mental health outcomes which may not consistently correlate with those of women who have experienced violence outside of an intimate relationship^15 33 34^ and that conflict-related violence can have long-lasting impacts that need to be should be addressed in the post-conflict period. Programmes to prevent and respond to GBV against women in these settings must, therefore, be developed to effectively address all forms of violence and be able to tailor services accordingly. Services and research should consider how to identify, measure and treat the impact of intimate partner violence including emotional abuse and controlling behaviours that may not be captured in current tools that are limited to physical and sexual violence. GBV services should also be integrated within programmes that target the wider population to allow for improved referrals pathway between GBV case management and other services including safe housing, legal advice and financial support. Task-sharing is one strategy that can be used to improve acceptability, accessibility and scale-up of services to refugee survivors of violence in a camp context.^35 36^ Further evaluation of preventive programmes which engage the community or intimate partners must also be undertaken to understand the most effective approach to tackling harmful normative beliefs that perpetuate further violence.^37^

Finally, there remain large gaps in the evidence-base for GBV programming in conflict-affected settings and subsequent research must endeavour to capture a greater breadth of the GBV experienced in these settings, including controlling behaviours, discrimination from communities and abuse from in-laws as well as explore the impact of the continuum of violence on women’s lives after the conflict.

**What is already known on this subject**

- There is growing evidence on the interrelationship between displacement due to conflict, violence and persecution, gender-based violence and mental health among refugee women. In humanitarian settings, identifying subgroups of women at higher risk of mental health may help to target services to those with the greatest need. However, there remains limited evidence on the differences in mental health outcomes among refugee women who have experienced violence in a refugee camp setting.

**What this study adds**

- More than half of the study population reported symptoms of probable post-traumatic stress disorder (PTSD) or severe/moderate depression or anxiety suggesting refugee women who experience violence in conflict-affected settings or recent intimate partner violence may have a higher prevalence of mental health disorders in comparison to the wider refugee population. In humanitarian settings, identifying subgroups of women who are at higher risk of mental health problems may help to tailor support services for survivors of violence.
